# MetaRange.jl: A Dynamic and Metabolic Species Range Model for Plant Species

**DOI:** 10.1002/ece3.70773

**Published:** 2025-01-10

**Authors:** Jana Blechschmidt, Juliano Sarmento Cabral

**Affiliations:** ^1^ Center for Computational and Theoretical Biology Julius‐Maximilians‐Universität Würzburg Würzburg Germany; ^2^ Biodiversity Modelling and Environmental Change University of Birmingham Birmingham UK

**Keywords:** dynamic range model, Julia language, metabolic theory of ecology, process‐based model, species distribution

## Abstract

Process‐based models for range dynamics are urgently needed due to increasing intensity of human‐induced biodiversity change. Despite a few existing models that focus on demographic processes, their use remains limited compared to the widespread application of correlative approaches. This slow adoption is largely due to the challenges in calibrating biological parameters and the high computational demands for large‐scale applications. Moreover, increasing the number of simulated processes (i.e., mechanistic complexity) may further exacerbate those reasons of delay. Therefore, balancing mechanistic complexity and computational effectiveness of process‐based models is a key area for improvement. A promising research direction is to expand demographically explicit metapopulation models by integrating metabolic constraints. We translated and expanded a previously developed R metapopulation model to Julia language and published it as a Julia module. The model integrates species‐specific parameters such as preferred environmental conditions, biomass and dispersal ability with demographic rates (e.g., reproductive and mortality rates) derived from local temperature and biomass via the metabolic theory of ecology. We provide a simple application example for the model in which we illustrate a typical use case by predicting the future occurrence of *Orchis militaris* in Bavaria under different climate change scenarios. Our results show that climate change reduces habitat suitability overall, but some regions like the Franconian Forest and the Alps see increased suitability and abundance, confirming their role as refugia. Simulating metapopulation dynamics reveals that local population dynamics and dispersal are crucial for accurate predictions. For instance, increasing dispersal distance reduces overall abundance loss but also lessens population growth in refugia. This highlights the importance of measuring traits like dispersal ability to improve climate change forecasts.

## Graphical abstract

We present MetaRange.jl, a Julia‐based model for predicting plant species distributions under climate change by integrating species‐specific parameters with demographic rates derived from the metabolic theory of ecology. Using *Orchis militaris* in Bavaria as a case study, our results show that while climate change generally reduces habitat suitability, some regions act as refugia, highlighting the importance of local population dynamics and dispersal in forecasting species responses to environmental changes.

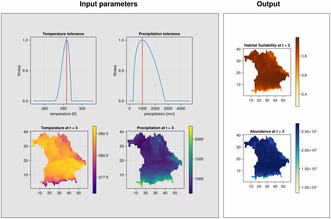

## 1 Background

Biodiversity has been globally on the decline due to human‐induced drivers, including climate and land‐use change, which has prompted urgent calls to mitigate this biodiversity crisis (Leclère et al. 2020; Mace et al. 2018; Díaz et al. 2019). To support mitigation actions, predictive tools are urgently needed (Díaz et al. 2019). To this task, the field of ecological modelling at the spatiotemporal extents necessary to tackle changes in species ranges continues to advance (Dormann et al. 2012; Cabral, Valente, and Hartig 2017; Urban et al. 2016). Process‐based modelling can be employed to estimate possible long‐term effects of conservation measures and to help identify biodiversity hotspots for conservation prioritisation (Sarmento Cabral et al. 2013). Moreover, process‐based modelling allows for predicting conservation‐relevant variables, such as abundance and range filling, and key dynamic trends such as migration rate, with a high level of transferability due to their incorporation of causal processes (Da Re et al. 2024). These are the advantages of process‐based models over commonly used phenomenological species distribution models (SDMs, see Zurell et al. (2016) for a comparison). Still, the development and application of process‐based range models remain modest due to the need for extensive ecological knowledge and computational feasibility.

With increasing computational power, the development of process‐based dynamic range models (DRMs) became more common; for example, Pagel and Schurr (2012), Bocedi et al. (2014) and Fallert, Li, and Cabral (2024). Ideally, a well‐parameterised DRM would not need to rely on SDMs to represent the potential range, but instead, directly formalise the relationship between the underlying processes with the environmental variables. These relationships are process‐specific response functions, for example, demographic response functions will determine parameters such as demographic transition rates (Schurr et al. 2012). However, a DRM with a large number of species‐specific parameters is more difficult to calibrate due to data deficiency. Furthermore, because these models are dynamic and may entail complex process interactions, simulations tend to be computationally demanding (i.e., increase in runtime), particularly over large numbers of individuals and grid cells. This is a central challenge for species risk assessments, as researchers are typically interested in the entire range of a species.

A well‐known DRM is Rangeshifter, which uses an individual‐based approach for simulating spatially explicit population dynamics (Bocedi et al. 2014) and was recently turned into an R package called rangeshiftR (Malchow et al. 2021). RangeshiftR can accommodate a high mechanistic complexity due to extensive species‐specific parameters, allowing a wide variety of research questions to be answered. Performance remains relatively fast, as most code is rather implemented in C++. Still, the large number of species‐specific parameters require extensive ecological knowledge for calibration (e.g., stage‐structured population matrices and dispersal behaviour). Hence, alternative models with a reduced number of parameters can further popularise process‐based range modelling.

One such alternative is metaRange, which integrates species‐specific parameters related to niche preferences, biomass and dispersal ability (Fallert 2021). An important feature of metaRange is that a high number of demographic parameters (e.g., reproductive and mortality rates, carrying capacity) are derived from just two species‐specific parameters, namely body temperature and body mass via the metabolic theory of ecology (MTE) (Brown et al. 2004) as demographic response function (Fallert 2021). The MTE links an organism's body mass and temperature to its metabolic rates. According to MTE, larger organisms have lower metabolic rates and high temperatures lead to higher biological rates, including metabolic (Brown et al. 2004) and demographic (Savage et al. 2004) rates. These two parameters, body mass and temperature, are much easier to find in the literature compared to demographic rates, particularly for plants and ectotherms where body temperature can be simplified to local environmental temperature. Additionally, using MTE for multiple demographic processes automatically accounts for metabolic trade‐offs, such as a higher body mass will impose simultaneously a lower reproductive rate and a higher survival rate (Savage et al. 2004). Finally, metaRange acts at population level, thus making it more easily applicable for larger spatiotemporal scales than individual‐based models.

In this study, we introduce MetaRange.jl—a metabolic‐based metapopulation model for range dynamics. It predicts species' occurrences based on fundamental ecological processes incorporating species‐specific traits and environmental local conditions. The MetaRange.jl model is written in Julia language, adapting to plant species the original R‐coded metaRange model for range dynamics of insect species (Fallert 2021). Julia is a relatively new open‐source and free programming language. It is of interest to scientists due to three main key features: speed, metaprogramming and abstraction (Roesch et al. 2023). While the most commonly used languages for ecological modellers, R and Python, often require to be partly re‐written in other languages, like C/C++, to be feasible for use, this is not the case for Julia. Due to its open‐source nature and encouraging community, Julia also has a quickly growing number of packages that facilitate complicated processes. Julia's main advantage (next to its simple syntax) is its speed. Ensuring a high speed enables us to simulate species at large spatiotemporal scales and high resolutions, while reducing computational demand, energy consumption and carbon footprint.

Here, we provide an in‐depth explanation on how to use the package with the integrated out‐of‐the‐box example. Furthermore, we use MetaRange.jl to describe the metapopulation and abundance dynamics of the orchid *Orchis militaris* for its Bavarian range, in Germany, until the year 2100 for different climate change scenarios, which will also provide an example of package utilisation.

## 2 Methods and Features

This Julia version is an extension for plant species of the metaRange model by Fallert (2021). MetaRange was originally published as an R package and intended to model the distribution of animals. It integrates spatially explicit metabolic, demographic and behavioural processes of virtual invertebrate species within a niche‐ and metabolic‐based approach considering species‐specific environmental preferences and MTE regulating demographic processes. Below we provide a short description of the model and extensions implemented to this version to make it applicable for plant species.

Parameters that are adjusted based on MTE depend on the given species‐specific reference value, local temperature and body mass of the species. For each grid cell in a given landscape, parameters are then calculated following Equation (1):

(1) $$Q=b_{0}\cdot m^{a}\cdot e^{\frac{-E}{k\cdot T}}$$

where *Q* is the respective rate that is to be modified, *b*_0_ is a normalisation constant (see Equation 2), *m* is the body mass of the plant individual, *a* is the exponent according to West, Brown, and Enquist (1997), *E* is the activation energy, *k* is the Boltzmann constant and *T* the temperature at the given grid cell.

(2) $$b_{0}=\frac{v}{m^{a}}\cdot e^{\frac{-E}{k\cdot T_{\mathrm{ref}}}}$$

The species‐ and temperature‐specific normalisation constant (Equation 2) can be calculated based on a species‐specific estimate or observation of the rate of interest *v* at a reference temperature *T*_ref_ (a species' optimum preferred temperature) and allows for the effects of body mass and temperature to be combined in Equation (1).

Environmental conditions are provided as a grid cell landscape with each grid cell containing a value that describes local conditions for this particular grid cell. It is required to provide a grid cell landscape containing temperature values, as these are crucial to the MTE calibrations for plants. A second parameter grid cell can be given, in case of our examples, it is annual precipitation. Furthermore, three species‐specific values per environmental niche parameter must be set for initialisation: minimum, maximum and optimum of the respective niche variable. From these values, the habitat suitability of each grid cell is calculated following Equation (3) derived from (Yin et al. 1995).

(3) $$h=\frac{v_{\max}-v_{\mathrm{env}}}{v_{\max}-v_{\mathrm{opt}}}\cdot {\frac{v_{\mathrm{env}}-v_{\min}}{v_{\mathrm{opt}}-v_{\min}}}^{\left(\frac{v_{\mathrm{opt}}-v_{\min}}{v_{\max}-v_{\mathrm{opt}}}\right)}$$

Here, *v*_max_ is the maximum tolerable value, *v*_min_ is the minimum tolerable value, *v*_opt_ is the optimal value and *h* is the resulting habitat suitability. This resulting suitability of a habitat is applied in Equation (5) and alters the carrying capacity of the respective habitat. The environmental suitability is often also estimated in popular correlative SDMs.

It is possible to consider more than two environmental niche variables to predict species' occurrences, such as nutrient content of the soil, etc. This input has to be in the same format as all other environmental variables (i.e., a .csv file with one value per grid cell). However, these values need to already be given as suitability, for example, by applying Equation (3), with the values ranging from zero to one. The full habitat suitability is calculated via multiplication or finding the minimum of all given environmental variables that affect the species. Each timestep, a population is updated following the Beverton–Holt population dynamics model according to Equation (4), which is different from the original metaRange R version. The original Ricker population dynamics model may also be used.

(4) Nt+1=Nt⋅r1+r⋅NtKh⋅r⋅mr−m+Nt−Nt⋅m

(5) $$K_{h}=K\cdot h$$

Here, *N*_*t*_ is the population abundance at timestep *t*, *r* is the species‐specific reproduction rate and *m* is the species‐specific mortality rate. The *K*_*h*_ is the species‐specific carrying capacity *K* multiplied by the local habitat suitability *h* (see Equation 5). The resulting *N*_*t*+1_ is the population abundance at timestep *t* + 1.

After reproduction according to (4), the produced seeds are dispersed from its original grid cell using a negative exponential dispersal kernel (6), with mean and maximum dispersal distances according to the species' traits. Due to this order of demographic processes, *N*_*t*+1_ includes recruitment from dispersal at timestep *t*.

(6) p=12⋅π⋅α2⋅e−rα

Here, *p* is the probability of dispersal, *2a* is the species‐specific mean dispersal distance and *r* is the distance from the origin cell. Note that the maximum dispersal distance must always be at least 1 for individuals to disperse at all. The mean dispersal distance can be smaller than one.

As a last step, offspring are recruited in new grid cells. Recruitment follows a Poisson distribution (7) if set to be stochastic to include demographic stochasticity (lambda being the dispersed seeds) and ensuring that abundance remains an integer. Abundance after recruitment will simply be rounded down if it is set to be deterministic.

(7) $$p_{\lambda }\left(k\right)=\frac{\lambda^{k}}{k!}e^{-\lambda }$$

### 2.1 Stochasticity

**FIGURE 1:**
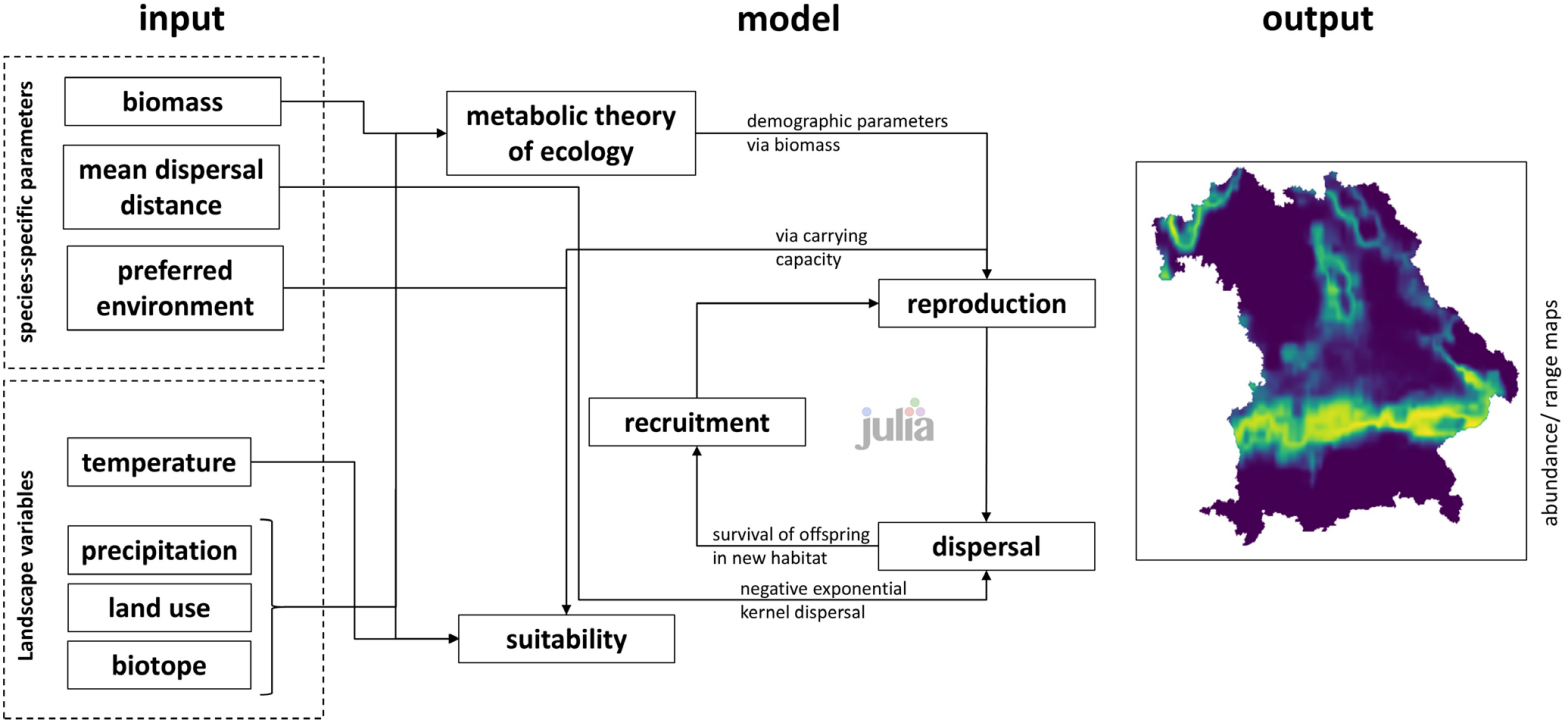
Flowchart of integrated processes, their input and their output within MetaRange.jl.

The model can consider stochastic effects for all or for selected processes. For this, stochasticity can be switched on and off by providing true/false arguments for the respective processes in the configuration file (Figure 1).

## 3 Examples

### 3.1 Example 1: ‘Out of the Box’ Experiment

To facilitate model understanding, we present a default experimental setup without input landscape but that creates and uses a random neutral landscape. This ‘Out of the box’ experiment can be used to ensure that the model runs and to familiarise with the output the model produces.

This experiment uses default experimental setup and species‐specific parameter values, which cannot be modified. As for environmental data, the experiment uses a random 20×20 grid cell landscape with only temperature and precipitation data. The random landscape was created using the NeutralLandscapes.jl Julia package. NeutralLandscapes.jl is a port of the respective Python package (Etherington, Penelope Holland, and O'Sullivan 2015) and can be found on GitHub. Temperatures have a mean value of 293.15 K with a standard deviation of 2.5 K, while precipitation has a mean of 500 mm year^−1^ with a standard deviation of 100 mm year^−1^. The experiment simulates 25 timesteps.

This experiment can be executed by assigning a name for the simulation struct (here SD, an abbreviation for Simulation Data) with the function SD = demo_input(). This will parse the default values and the created data into the correct format required by the model and store it in a mutable SimulationData struct named SD. The same SD can now be used to run the implemented processes, being modified during the simulation as indicated by the !:

run_simulation!(SD)

The model will simulate the metapopulation dynamics with explicit local population dynamics constrained by metabolic relationships of its parameters and with populations connected by kernel‐based dispersal, printing at the terminal its progress at every timestep. When the simulation is completed, it will also print the time elapsed. The simulation output stored in the SimulationData struct can be accessed by calling its properties (see Figure S1 for the structure of SD). For example, to investigate the abundances of the first species modelled in timestep 4, type

SD.species[1].output.abundances[:,:,4]

to access the abundance array (check further output options in Table B4 of Box 1). MetaRange.jl also comes with predefined plotting functions (see Section 2.2.2).

BOX 1 **Installing and running the model.**

MetaRange.jl can be used by modellers and empiricists, who may not be familiar with process‐based models. We provide an extensive guide on the model's repository page (https://janablechschmidt.github.io/MetaRange.jl/dev/), but starting instructions are provided in this box. To instal the model, one can use the inbuilt Julia package manager and execute.

julia> import Pkg

julia> Pkg.add(" https://github.com/janablechschmidt/MetaRange.jl.git ")

This will download and instal the MetaRange Julia package with all required dependencies (Table B1).

**TABLE B1** Packages required by MetaRange.jl and what they are used for.

**Table:** 

Package name	Application
DelimitedFiles.jl	Loading and saving data in .csv format
CSV.jl	Loading and saving data in .csv format
DataFrames.jl	Loading and saving data in .csv format
Distributions.jl	Stochastic effects
Random.jl	Stochastic effects
Dates.jl	Keep track of simulation time
CairoMakie.jl	Plot the output of the model

Executing the model consists of two main functions: read_input() and run_simulation!(). The argument provided to read_input() must be the pathname to the configuration file (see repository for a file example), which provides the parameter values for the simulation experiment. Configurations can be found in Table B2.

**TABLE B2** Model simulation configuration parameters and their respective default, type and description. Parameters with no default must be provided by the user, namely the path to the configuration file and environmental parameter (temperature and precipitation) files.

**Table:** 

Parameter	Default	Type	Description
experiment_name	default	String	Name of experiment
config_dir		String	Path to the configuration file
output_dir	./output/	String	Path to output folder
species_dir	./species/	String	Path to the species folder relative from the configuration file. The default assumes a species folder in the same folder as the configuration file
environment_dir	./environment/	String	Path to the environment folder relative to the configuration file. The default assumes an environment folder in the same folder as the configuration file
input_backup	false	Bool	If ‘true’, input files will be copied to output folder
temperature		String	Path to temperature input file(s) relative to environment directory. Can be a folder containing ordered CSV files or a single CSV file
precipitation		String	Path to precipitation input file(s) relative to environment directory. Can be a folder containing ordered CSV files or a single CSV file
restriction	nothing	String	Path to restriction input file(s) relative to environment directory. Can be a folder containing ordered CSV files or a single CSV file
env_attribute_mode	minimum	String	Mode for environment attributes. Can be multiplication or minimum
env_restriction_mode	minimum	String	Mode for restriction attributes. Can be multiplication or minimum
timesteps	20	Int	Number of timesteps to run
randomseed	42	Int	Random seed for simulation
reproduction_model	Beverton	String	Sets which Reproduction equation will be used. Can be defined as ‘Beverton’, ‘Ricker’ or ‘RickerAllee’
use_metabolic_theory	true	Bool	If ‘true’ the Metabolic Theory of Ecology (Brown et al. 2004) will be used to calculate reproduction and mortality rates, carrying capacity, allee threshold
use_stoch_num	false	Bool	If ‘true’ the number of individuals surviving dispersal will include stochasticity via Poisson (7)
initialise_cells	habitat	String	Sets where the species will be initialised. Can be defined as ‘habitat’, ‘all’, ‘random’

The function read_input() will read all the input (the file with experimental configuration, species‐specific parameter value and environmental variables), parse it into the correct data types and provide an initialised SimulationData struct as output. This is a specifically declared data type that holds all information about the simulation experiment, including the species parameters, the environmental parameters and the simulation settings (see repository or appendix for the structure of the SimulationData struct).

This SimulationData struct is to be used as input for the run_simulation!() function. This function will calculate the species' abundances and distribution for the provided landscape and timeframe. This will modify the SimulationData struct and store the results within (Table B3).

**TABLE B3** Output parameters of the model and their respective names to access them directly.

**Table:** 

Output Variable	Variable name
Abundances	SD.species[1].output.abundances
Habitat suitability	SD.species[1].output.habitat
Carrying capacity	SD.species[1].output.carry
Reproduction rate	SD.species[1].output.growrate
Mortality rate	SD.species[1].output.bevmort

We provide a function to save the output of the simulation in one single .tsv file: save_all(SD). The structure of the output file is ‘t, x, y, value, parameter’. For example, the first row will be ‘1, 1, 1, 0, abundance’, meaning at timestep 1, in the grid cell (1,1), the abundance was zero. It is also possible to write custom functions to save any parameters of interest by simply referring to the respective entry in the SimulationData struct.

MetaRange.jl also comes with several predefined plotting functions to visualise the results (Table B4).

**TABLE B4** Plotting functions and their respective output.

**Table:** 

Function name	Output
plot_abundances(SD)	Plots total abundance and carrying capacity over time
img(SD, output, t)	Shows map of selected output and timestep
gif(SD, output)	Creates a gif of the selected output over time
img_complex(SD, t)	Shows temperature tolerance, precipitation tolerance, precipitation, temperature, abundances and habitat suitability for a selected timestep
gif_complex(SD)	Shows temperature tolerance, precipitation tolerance, precipitation, temperature, abundances and habitat suitability as a gif over time

### 3.2 Example 2: Future Range of *Orchis militaris* in Bavaria

We apply MetaRange.jl to predict the distribution of the orchid *Orchis militaris* in Bavaria based on environmental and functional data. MetaRange.jl comes with two example folders containing all required input files to run a simulation: *Example1_Static_Environment* and *Example2_Environmental_Change*. Both contain species‐specific parameters, a configuration file and environmental files. Here, we show results from the climate change experiment (i.e., using *Example2_Environmental_Change*). The environmental data were given by a climate change scenario from 2020 to 2100 of the v2.1 of the climatology database CHELSA (Karger et al. 2017; Brun et al. 2022). We selected projections for climate change under the SSP3‐RCP7 using the gfdl‐esm4 model for the region of Bavaria. To keep the example fast and reproducible for all machines, data were downscaled by a factor of 10 to reduce the size of the files (grid cell of 100 km^2^) and the amount of time for global warming was accelerated to 40 years, with an onset of climate change in year 11. The original dataset spans from 1979 to 2100. Functional traits were translated into values for the species‐specific parameters (Table 1, also in the GitHub repository in the ‘examples’ folder). By an onset of climate change in year 11, we have a 10‐year burn‐in period during which the environmental conditions are kept stable. This allows the initialised metapopulation to reach a dynamic equilibrium before the onset of climate change. The length of the burn‐in period and the strength of its effects depend on the chosen method of initialisation. Here, populations are initialised in all suitable grid cells with population sizes corresponding to habitat suitability, leading to only small changes in distributions based on dispersal. When populations are initialised randomly in the landscape or in all grid cells regardless of habitat suitability, we recommend burn‐in periods to be longer before starting experiments.

**TABLE 1:** Species‐specific traits, the corresponding argument name in the model, value and source for *Orchis militaris* as used in the application example. Note that temperature may be given in Celsius or Kelvin.

Trait	Parameter	Value [unit]	Source
Carrying Capacity	carry	266,667 ind km^−2^	Henneresse, Wesselingh, and Tyteca (2017)
Reproduction Rate	growrate	1.15	https://compadre‐db.org/Species/46858
Mortality Rate	bevmort	0.44	https://compadre‐db.org/Species/46858
Maximum Dispersal Distance	max_dispersal_dist	1 grid cell	0.5 km (Vittoz and Engler 2007)
Mean Dispersal Distance	mean_disperal_dist	0.0005 grid cells	0.005 km (Vittoz and Engler 2007)
Biomass	mass	23.82 g	Caliskan, Kurt, and Odabas (2020)
Maximum Temperature	upper_limit_temperature	15.28°C	GBIF/wc2.1 Europa occurrences
Minimum Temperature	lower_limit_temperature	−3.36°C	GBIF/wc2.1 Europa occurrences
Optimum Temperature	optimum_temperature	10.2°C	GBIF/wc2.1 Europa occurrences
Maximum Precipitation	upper_limit_precipitation	2772	GBIF/wc2.1 Europa occurrences
Minimum Precipitation	lower_limit_precipitation	297	GBIF/wc2.1 Europa occurrences
Optimum Precipitation	optimum_precipitation	1000	GBIF/wc2.1 Europa occurrences

To reproduce this experiment, one can execute:

using MetaRange

SD = read_input(“your/path/to/Example2_Environmental_Change/configuration.csv”)

**FIGURE 2:**
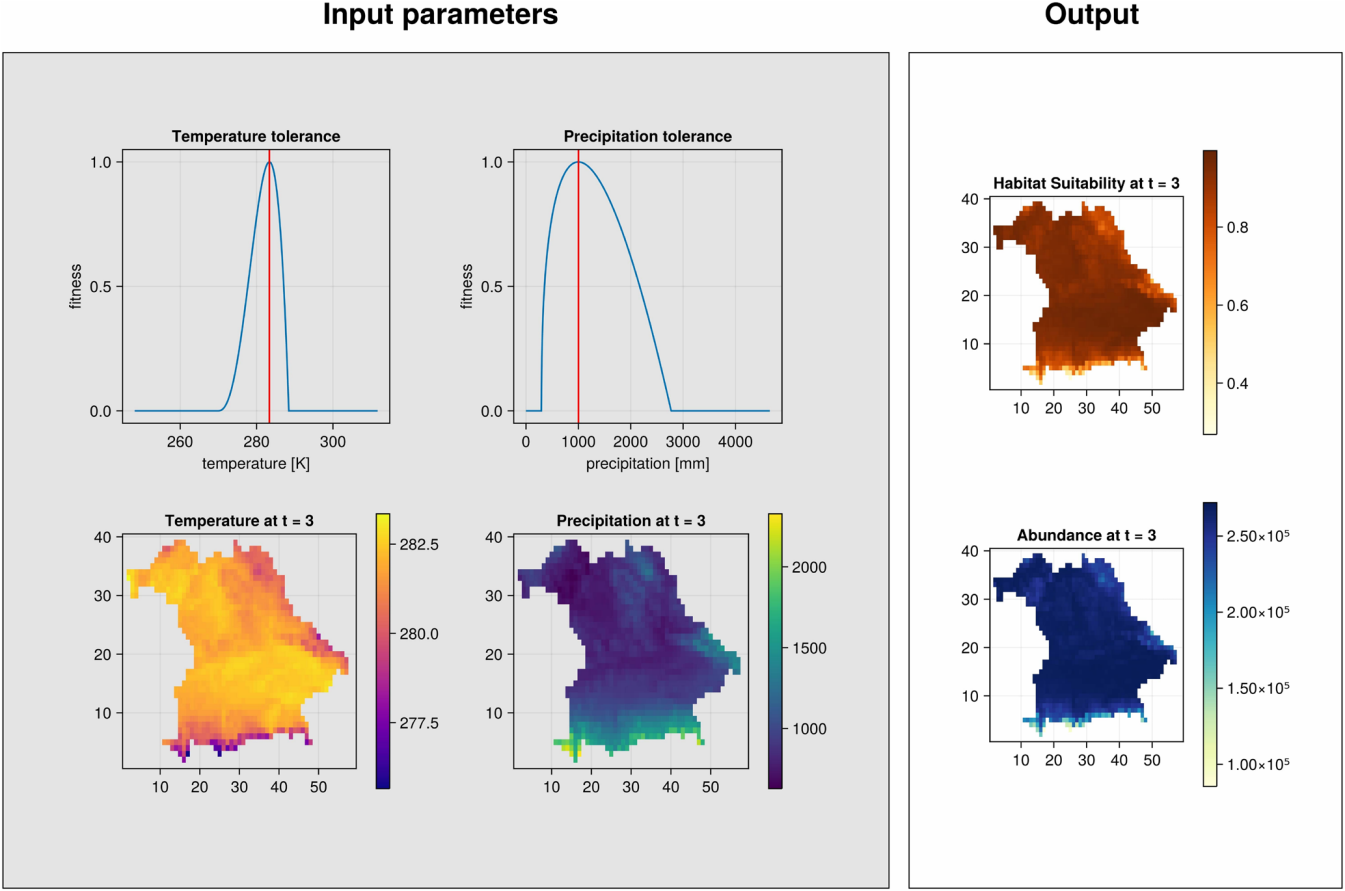
Input and output of the model at timestep *t* = 3. The left box displays certain model inputs (from top left, clockwise: Species‐specific temperature tolerance with optimum indicated by red line, species‐specific precipitation tolerance with optimum indicated by red line, average annual precipitation at selected timestep, average annual temperature at selected timestep). The right box shows the output of habitat suitability (top) and of abundances (bottom) at the selected timestep.

The output can be plotted by referring to the respective entries in SD, but there are also several plotting options provided by MetaRange (see Documentation). For example,

plot_all(SD,4) will recreate Figure 2.

**FIGURE 3:**
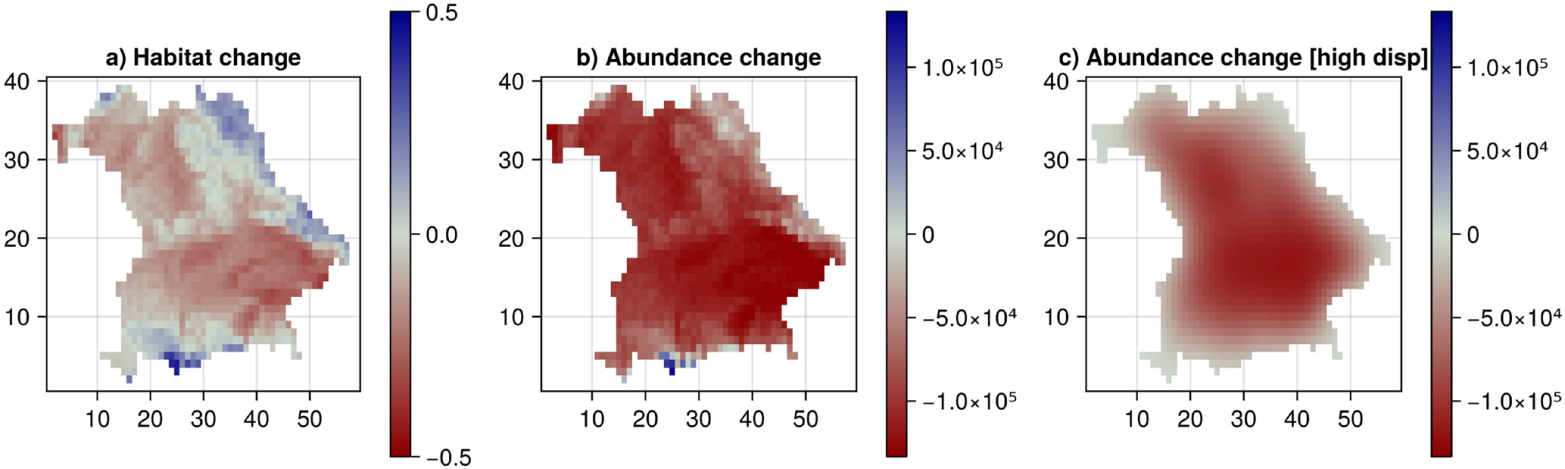
Changes in abundance and in habitat suitability. (a) Absolute changes in habitat suitability from timestep *t* = 10 to timestep *t* = 40. Losses are indicated in red, gains in blue. Areas without change are indicated in grey. (b) Absolute changes in abundances in number of individuals. (c) Absolute changes in abundances in number of individuals when artificially increasing the dispersal ability to a mean distance of 3 km.

We identified the geographical regions with abundance change (Figure 3 below).

For *Orchis militaris*, both abundance and habitat suitability change as the climate changes (Figure 3a,b). We compare here the abundances of timestep *t* = 40 (end of simulation) with the abundances of timestep *t* = 10 (end of burn‐in period). Results show that habitat suitability drops almost everywhere, but remains largely stable in the Franconian Forest (middle to upper North/East) and increases in more elevated regions in the alps (South), the Bavarian and Upper Palatinate Forests (Northeast/East) and in the Rhoen (Northwest) (Figure 3a). The abundance of the species increases in these areas, while it decreases in the rest of Bavaria (Figure 3b). Those areas of abundance increase coincide with protected areas (biosphere reserve Rhoen, several national parks and nature reserves in the alps, nature reserve and park upper palatinate forest, nature park franconian forest), whose role as key refuge areas in conservation efforts is then confirmed in our experiment.

It is important to highlight that the local population dynamics and dispersal between habitats are central to refining predictions beyond changes in habitat suitability. To demonstrate that, we artificially increase the mean dispersal distance of *Orchis militaris* from 0.005 to 3 km, which results in a less severe abundance loss across the landscape, but also in a less pronounced population growth in predicted refugia (Figure 3c). This shows that increased dispersal ability (often taken as a full‐ or global dispersal scenario in SDM‐based assessments) may be misleading in forecasting metapopulation response to climate change, as it increases the role of source‐sink dynamics while decreasing important mass effects. This also underlines the necessity to adequately measure functional traits that are relevant to range models, such as mean dispersal ability.

## 4 Discussion

Process‐based spatially explicit SDMs can play a huge role in determining areas of interest for conservation measures for important species. Predictions of abundances of populations can be forwarded to policy makers and thus have a real impact on future biodiversity. DRMs have proven to be more adequate than SDMs for range dynamics under changing environments, but they rely on species‐specific ecological knowledge to calibrate parameters and can be very computationally expensive (Zurell et al. 2016; Dormann et al. 2012). MetaRange.jl tackles this issue by simplifying the number of parameters by applying the Metabolic Theory of Ecology to derive demographic rates (Savage et al. 2004; Cabral and Kreft 2012; Cabral et al. 2019; Leidinger, Vedder, and Cabral 2021).

Developing metaRange into a Julia package expands the reach of the model. Julia is predicted to increasingly be used by biologists/ecologists (Roesch et al. 2023). The key advantage of julia is a high computational efficiency, similar to C++, while still maintaining a user‐friendly syntax (Roesch et al. 2023). This will enable many developments in julia‐based range models, such as an increase in resolution and amount of simulated populations as well as further mechanistic development of the dynamics (e.g., integrating biotic interactions and evolution). Our package MetaRange.jl is written in a very beginner‐friendly way, with extensive documentation, tutorials and a guide directed at newcomers in computer modelling in general. This opens up the world of ecological modelling to inexperienced users and can help connect empirical research with modelling approaches.

### 4.1 Limitations and Perspectives

Because real‐world applications of MetaRange.jl have simulated fairly big landscapes (> 2500 grid cells), we simply implemented absorbing edges into the code. However, in our artificially increased dispersal distance scenario (Figure 3c), some edge effects can be seen, where population sizes at the border have dropped to zero due to the absorbing effect of the boundary. Hence, for smaller landscapes or organisms with large dispersal distances, it might be worth implementing reflecting edges as a future improvement to MetaRange.jl. Nevertheless, under absorbing edges, edge effects can be avoided in an area of focus if the simulation arena considers a buffer rim around the focus area that is larger than the maximum dispersal distance.

Metarange.jl is only able to simulate one single species at a time. For the future, we plan on expanding the model to be able to include biotic interactions, for example, via a shared resource, to make model predictions even more accurate.

One caveat of using MetaRange for plants is that even though there are a number of plant trait databases (Kattge et al. 2011; Kleyer et al. 2008; Salguero‐Gómez et al. 2015), relevant ecological data on plant species is scarce. This is especially true for traits such as dispersal ability (travel distance in metres), and physiological niche preferences (which may be known for model or crop plant species, but not generally for many plants). Therefore, many parameter values must be estimated. While it is possible to empirically research many physiological traits (e.g., temperature preferences) of small animals (e.g., grasshoppers; Fallert 2021), doing so for plants requires extensive glasshouses or common garden experiments. Potentially for a plant species' tolerable temperature range, they can be at least partially inferred via occurrence data like they would in a typical SDM. To do so, one can extract local environmental conditions at coordinates where species occur, for example, using the GBIF database (*GBIF: The Global Biodiversity Information Facility* 2024). Expanding the geographic scope beyond the region of interest—for instance, considering European occurrences when modelling a German species—provides a more comprehensive view. This approach helps avoid modelling the realised niche and instead captures the actual niche (Hutchinsonian shortfall) (Scherrer et al. 2021). There are current projects which address the issue of data deficiencies in other areas, for example, VecTraits (https://www.vectorbyte.org/blog/introducing‐vectraits) for functional trait data of vectors, or the Add‐My‐Pet portal (https://www.bio.vu.nl/thb/deb/deblab/add_my_pet/) for animal energetics.

There are several directions for future model development. First, we plan on enabling MetaRange.jl to handle multiple species at once and include biotic interactions between species via a shared resource (already available with the R version; Fallert, Li, and Cabral 2024). Next, it might be helpful to increase flexibility in depicting the ecological performance along niche axes by allowing for other environmental conditions tolerance curves other than the one based on Yin et al. (1995) and Yan and Hunt (1999), for example, by making it possible to use a simple Gaussian curve. Furthermore, while we do believe the edge effects to be neglectable for most applications, implementing reflecting edges (where dispersing individuals get ‘reflected’ back into their cell of origin) or closed torus (where dispersing individuals re‐enter the landscape on the opposite side) could increase potential experimental designs. Different edge rules may facilitate the implementation of ecologically meaningful scenarios such as continuous plots within vegetation (e.g., via reflecting or periodic edges) or isolated habitat patches or island‐like systems (e.g., absorbing edges). It might also be interesting to implement further demographic functions, for example, including allee effects (but see Fallert, Li, and Cabral 2024). Lastly, a useful expansion of MetaRange.jl would be to allow it to work with Raster data instead of transforming all input and output to CSV files. Julia only recently started handling Raster data, thus such development can be well achieved in the near future.

Further guidelines for experimental designs and configurations can be found in the package Github project page (https://janablechschmidt.github.io/MetaRange.jl/dev/).

While there are several directions for future development of the package, the current version of MetaRange.jl is already a powerful tool to predict species' future ranges as demonstrated in our examples. This increases the available palette of options for the much‐needed DRMs, hopefully attracting more users from empirical fields and among stakeholders. A particularly important use case may be identifying hotspots for conservation areas for certain species, and informing stakeholders so that conservation measures may be put into place.

## Author Contributions

**Jana Blechschmidt:** conceptualization (equal), software (lead), visualization (lead), writing – original draft (lead), writing – review and editing (equal). **Juliano Sarmento Cabral:** conceptualization (equal), supervision (lead), writing – review and editing (equal).

## Conflicts of Interest

The authors declare no conflicts of interest.

## Supporting information


Appendix S1

## Data Availability

All data and code are available in the MetaRange.jl repository on GitHub (https://github.com/janablechschmidt/MetaRange.jl) and on Dryad at doi:10.5061/dryad.612jm64d9.
