# Supplementary material for: MetaRange.jl: A Dynamic and Metabolic Species Range Model for Plant Species
Source: Ecol Evol. 2025 Jan 10;15(1):e70773. doi: 10.1002/ece3.70773 (PMC11724151; doi:10.1002/ece3.70773)
Supplement: Supplementary file 1 — Appendix S1 [file ECE3-15-e70773-s001.docx]

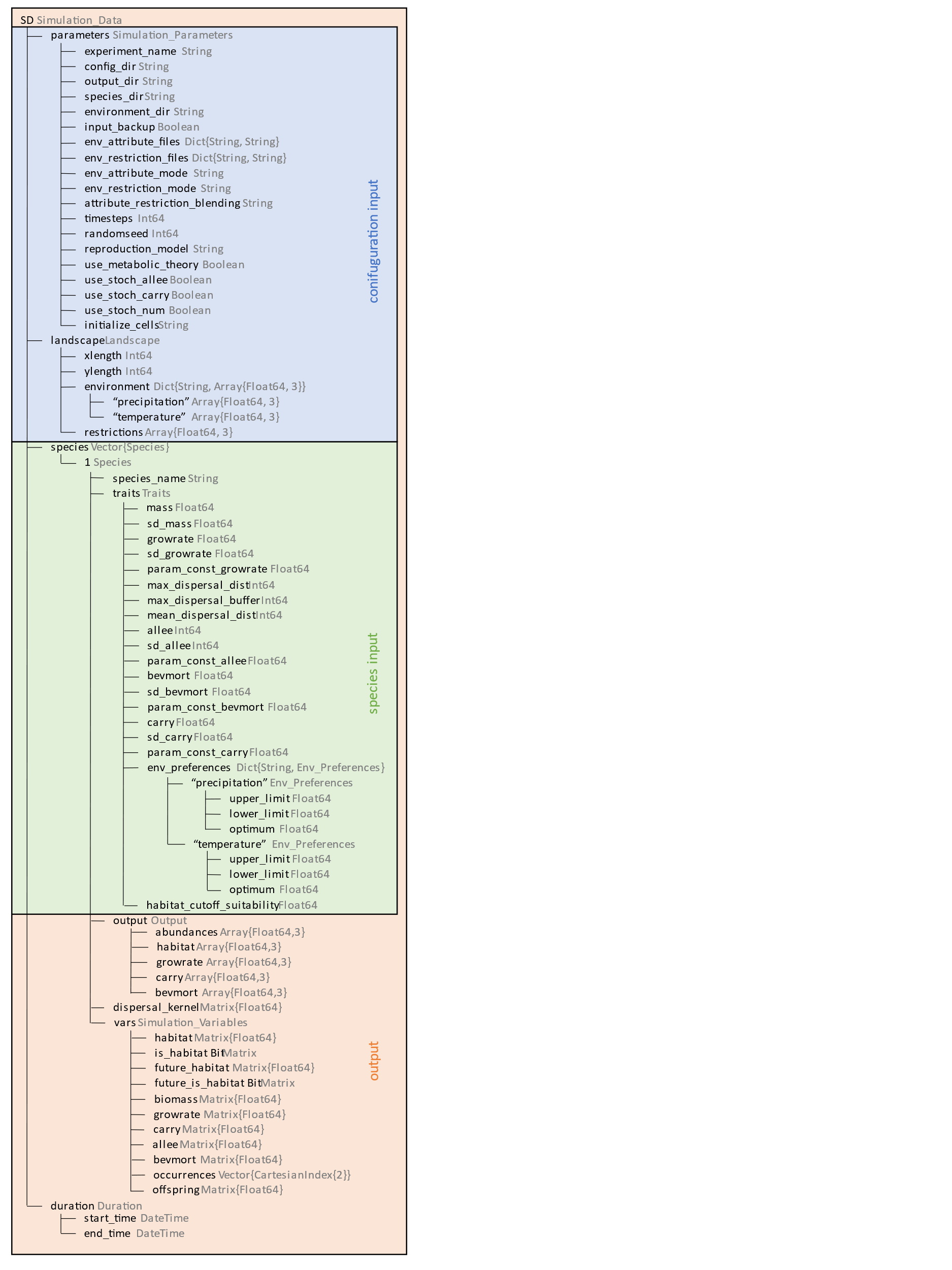


Figure SX1: Structure of the SimulationData struct. It includes all configuration and species input variables as well as the computed output.

Note on Species Trait Data:

Environmental preferences were extrapolated from environmental conditions at the place of occurrence in Europe. Occurrence data was acquired with the occ_data() function of the rgbif package. We included all occurrences with a coordinate within Europe. The databank was accessed on 18.05.2023.
